# 

**Published:** 1878-02-20

**Authors:** 


### CONTENTS.

### ORIGINAL AND SELECTED ARTICLES.

Does Hydrate of Chloral sometimes Act with Unusual Energy when Administered in Conjunction with a Preparation of Opium? By S. S. Shields, M.D., Crawfordville, Ga. 33
On the Use of Opium in Congestion; by A B. Loving. M.D. .... 35
Hysterica Epileptica with Unusual Complications; by John Coston. M.D., of Ala. ... 37
Fissure of the Rectum; by Erskine Mason. M. D .... 38
Corneal Ulcers; by I. R. L. Hardesty, M.D., Wheeling, W. Va .... 40
Bectal Alimentation .... 42
Unusual Action of Anæsthetics .... 44
Œnothera Biennis in Mucous Inflammations; By James F. Sulliva , M.D .... 46
Atlanta Medico-Chirurgical Association .... 48
Utero-Gestation Table .... 49

### ABSTRACTS AND GLEANINGS.

Graves' Disease, 50; Listers Antiseptic Method. 51. Treatment of Chronic Gonorrhœa, 52; Warts, 53; Urticaria and Quinine, 53; New Method of Reducing Dislocations of the Shoulder, 54; Hemorrhoids, 54; Viburnum, 55; Shall Medical Witnesses Receive a Proper Compensation? 55; Elephantiasis of the Penis, 55; American Gum Arabic, 56; Umbilical Hernia. 56; Instantaneous Cure of Hydrocele, 56; Tracheotomy, 56; Gutta Percha Tissue, 57; Cota Bark in Diarrhœa and Rheumatism, 57; Folicular Stomatitis, 57.

### PRACTICAL NOTES AND FORMULÆ.

Diseases of the Teeth, 58; Godfrey's Cordial, 58; Condition Powder for Horses, 58; Chilblains, 59; Hysteria, 59; Excellent Podophyllin Pill, 59; Black Hair Dye without Silver, 59; Syr. Citrate of Iron and Silver, 59; Cyanide of Zinc, 59; Arsenical Paste, 60; Turpentine in Typhoid Fever, 60; Dalby's Carminative, 60; Elixir Valerean, 60; Diabetes, 60; Menorrhagia, 60; Churchhill's Tinct. Iodine, 60; A Substitute for Litmus Paper, 60.

### SCIENTIFIC ITEMS.

Medical Progress in 1877, 61; Liquefaction of Gases, 61.

### EDITORIAL AND MISCELLANEOUS.

Crowded, 62; Elixir Guarano, 62; Envy, 63; Books and Drug Men, 63; Tribute to Dr. P. F. Eve, 63; The Sample Copy Man, 64; Book Notices, 64.

